# Cardio-metabolic and socio-demographic risk factors associated with dependency in basic and instrumental activities of daily living among older Iranian adults: Bushehr elderly health program

**DOI:** 10.1186/s12877-021-02124-x

**Published:** 2021-03-09

**Authors:** Kazem Khalagi, Akram Ansarifar, Noushin Fahimfar, Mahnaz Sanjari, Safoora Gharibzdeh, Farshad Sharifi, Gita Shafiee, Ramin Heshmat, Iraj Nabipour, Bagher Larijani, Afshin Ostovar

**Affiliations:** 1 Osteoporosis Research Center, Endocrinology and Metabolism Clinical Sciences Institute, Tehran University of Medical Sciences, Tehran, Iran; 2 Department of Epidemiology, School of Public Health, Iran University of Medical Sciences, Tehran, Iran; 3 Osteoporosis Research Center, Endocrinology and Metabolism Clinical Sciences Institute, Tehran University of Medical Sciences, Tehran, Iran; 4 Department of Epidemiology and Biostatistics, Pasteur Institute of Iran, Tehran, Iran; 5 Elderly Health Research Center, Endocrinology and Metabolism Population Sciences Institute, Tehran University of Medical Sciences, Tehran, Iran; 6 Chronic Diseases Research Center, Endocrinology and Metabolism Population Sciences Institute, Tehran University of Medical Sciences, Tehran, Iran; 7 The Persian Gulf Marine Biotechnology Research Center, the Persian Gulf Biomedical Sciences Research Institute, Bushehr University of Medical Sciences, Bushehr, Iran; 8 Endocrinology Research Center, Endocrinology and Metabolism Clinical Sciences Institute, Tehran University of Medical Sciences, Tehran, Iran

**Keywords:** Cardio-metabolic, Socio-demographic, Risk factor, Disability, Basic activities of daily living, Instrumental activities of daily living, Geriatrics, Iran

## Abstract

**Background:**

Iran’s population is aging. Disability is a major public health problem for older adults, not only in Iran but all over the world. The purpose of this study was to investigate the relationship between cardio-metabolic and socio-demographic risk factors and disability in people 60 years and older in Iran.

**Methods:**

The baseline (cross-sectional) data of 2426 samples from the Bushehr Elderly Health (BEH) program was included in the analysis. The participants were selected through multi-stage random sampling in Bushehr, southern Iran. Socio-demographic characteristics, as well as the history of diabetes and other chronic diseases, and smoking were measured using standardized questionnaires. Anthropometric measurements and laboratory tests were performed under standard conditions. Dependency was determined by the questionnaires of basic activities of daily living (BADL) and instrumental activities of daily living (IADL) using Barthel and Lawton scales respectively. Multiple logistic regression was used in the analysis.

**Results:**

Mean (Standard Deviation) of the participants’ age was 69.3 (6.4) years (range: 60 and 96 years), and 48.1% of the participants were men. After adjusting for potential confounders, being older, being female (OR (95%CI): 2.3 (1.9–2.9)), having a lower education level, a history of diabetes mellitus (OR: 1.4 (1.2–1.7)) and past smoking (OR: 1.3 (1.0–1.6)), and no physical activity (OR: 1.5 (1.2–1.9)) were significantly associated with dependency in IADL. Also, being older and female (OR: 2.4 (1.9–3.0)), having a lower education level, no physical activity (OR: 2.2 (1.6–2.9)) and daily intake of calories (OR: 0.99 (0.99–0.99)) were associated with dependency in BADL.

**Conclusion:**

Dependency in older adults can be prevented by increasing community literacy, improving physical activity, preventing and controlling diabetes mellitus, avoiding smoking, and reducing daily calorie intake.

**Supplementary Information:**

The online version contains supplementary material available at 10.1186/s12877-021-02124-x.

## Background

According to the International Classification of Functioning, Disability, and Health (ICF), disability is defined as disorders and limitations of activity and participation. Disability is the result of an interaction between illness and personal and environmental factors (e.g., negative attitudes, inability to use the transportation system and inadequate public facilities, and inadequate social support) [1]. The Barthel activities of daily living (ADL) and the Lowton instrumental activities of daily living (IADL) indices are standard tools for measuring disability [2, 3]. In recent years, the burden of disability increased by 52% worldwide. According to the results of a population-based study in Tehran, the prevalence of disabilities in individuals aged ≥60 years was 11% in 2011 [4]. In another study in 2012 in Iran, 13.2% of women and 12.6% of men were dependent on others for at least one daily activity [5]. Almost 80% of these disabilities are the result of non-communicable diseases. According to the Global Burden of Disease (GBD), diabetes was the fourth leading cause of disability in the world in 2017 [6]. Cardio-metabolic risk refers to risk factors that increase the chance of experiencing cardiovascular events such as heart attack or stroke. This concept includes traditional risk factors, such as age, sex, obesity, family history, hypertension, dyslipidemia (high LDL cholesterol, high triglycerides, and low HDL cholesterol), dysglycemia, and smoking, as well as emerging risk factors, such as abdominal obesity (measured by waist circumference), inflammation as measured by high-sensitivity C-Reactive Protein levels, insulin resistance, lack of consumption of fruits and vegetables, psychosocial stress, and sedentary lifestyle [7]. These risk factors can increase the likelihood of disability, either directly or by increasing the risk of cardiovascular events.

Disability risk factors vary across communities and regions of the world. According to a study in Australia, disability was positively associated with smoking, obesity, diabetes, and being woman [8]. In another study in the Netherlands, the most important predictors of disability in old age were previous disability and age. Other factors (such as sex, cognitive function, peer health score, obesity, hypertension, and joint pain) did not play a significant role in increasing disability [9]. In another study conducted in 6 middle and low-income countries (India, Ghana, South Africa, Mexico, Russia, and China), age, chronic diseases (such as hypertension, angina, stroke, diabetes, chronic lung disease, asthma, and arthritis) and depression were identified as the most important factors affecting disability. Other factors (such as sex, marital status, education, social capital, physical activity, BMI) either had no role or played a different role in societies [10].

In a study of the older adults in Spain, the hazard ratios (HRs) in the physical disability domain questionnaire were 1.14 to 1.52 for total mortality and 1.29 to 1.58 for cardiovascular diseases (CVD) compared to people with no disabilities. The researchers found that in older people with disabilities, physical activity reduces the risk of total death as well as death from CVD. The death rate in disabled people is similar to inactive people with no disability. The suggested mechanism is physical activity reduces obesity, sarcopenia, falls, and more. Physical activity also improves one’s social network, mood, and depression; all of them are associated with reduced morbidity and mortality [11].

Dhamoon et al. showed that although the maximum rate of disability due to stroke and myocardial infarction (MI) occurs during the stroke, the rate of disability continues to increase annually in people who experience these vascular events. This is even worse in people who have had a stroke [12]. Therefore, if the disability status is determined using self-report, it may consider the timing of the underlying disease.

Iranian population has quadrupled over the last six decades, while population growth has almost halved [13]. Also, from 1970 to 2010, life expectancy increased from 50.6 to 71.6 years for Iranian men and from 56.2 to 77.8 years for women. Iran is one of the countries that during this period has experienced significant improvements in life expectancy at birth in both sexes [14]. One of the most common problems in Iranian public health is the high prevalence of cardio-metabolic risk factors. For example, the results of a meta-analysis showed that about one-third of Iranians had metabolic syndrome. Besides, the prevalence of this syndrome increases with age [15]. According to Sadeghi et al., only due to aging, the CVD burden and DALY in the Iranian population will double in 2025 compared to 2005 [16]. The highest YLL, YLD, and DALY will be in people over 80 years of age [17]. The purpose of this study was to investigate the relationship between cardio-metabolic and socio-demographic risk factors and disability using basic and instrumental activities of daily living, in people 60 years and older in Iran.

## Methods

### Study design

In this study, a total of 2426 people from the baseline (second stage of the first phase) of a population-based prospective cohort study, the Bushehr Elderly Health (BEH) program [18, 19] were included in the analysis. The purpose of the BEH study, whose methodology has been described elsewhere [18, 19], is to investigate the incidence of non-communicable diseases and associated risk factors among people 60 years and older. The participants were selected through multi-stage stratified cluster random sampling in Bushehr, southern Iran [18]. The first stage of the first phase of the BEH program was implemented from March 2013 to October 2014. Prevalence of cardiovascular risk factors was investigated among 3000 men and women (participation rate = 90.2%) in this stage [18]. The second stage of the first phase of the study was conducted 2.5 years later on 2772 eligible persons from the first stage. The prevalence of musculoskeletal and cognitive diseases and their risk factors was investigated in this stage. Seventy-eight subjects eligible to participate in the second stage of the first phase, due to death, and 268 subjects due to loss to follow-up, did not attend at this stage (12.5%) [19]. Both stages of the first phase of the study are the baseline phase of the BEH program, and each stage has been focused on measuring the prevalence of the specific groups of non-communicable diseases and their risk factors [18, 19]. Follow-up of the non-communicable disease incidence in the enrolled subjects will be done in the next phases of the BEH program.

### Measurements outcomes

In this study, disability was measured by two questionnaires of Basic Activity of Daily Living (BADL) using Barthel scale [3], and Instrumental Activity of Daily Living (IADL) using Lawton [2] scale, through face to face interview with the participants by the trained questioners. The validity and reliability of these questionnaires were previously assessed in Iran and were at acceptable levels [20, 21]. The BADL questionnaire has 10 items including eating, bathing, urine control, toilet use, moving from bed to chair and vice versa, dressing, self-cleaning, stool control, climbing stairs, and ability to move on a flat surface. The IADL questionnaire has 8 items including the ability to use the phone, cooking meals, washing clothes, taking medication, shopping, housekeeping, transportation, and financial ability. These two questionnaires assess the degree of dependency of older adults. In the BADL questionnaire, the subjects with total scores of < 95 on the Barthel scale were considered as a dependent, and those with scores of 95 to 100 were considered as independent. The subjects with total scores of 0 to 7 of the IADL questionnaire were also defined as dependent and those with a score of 8 were defined as an independent.

### Cardio-metabolic and socio-demographic risk factors

In this study, we considered cardio-metabolic and socio-demographic risk factors as independent variables. Cardio-metabolic risk factors refer to risk factors that increase the chance of experiencing cardiovascular events, such as age, sex, obesity, hypertension, dyslipidemia (high LDL cholesterol, high triglycerides, and low HDL cholesterol), dysglycemia, smoking, abdominal obesity, lack of consumption of fruits and vegetables, and sedentary lifestyle.

Socio-demographic characteristics including sex, age, marital status, and education level, as well as information on cardio-metabolic risk factors including history of diabetes mellitus and hypertension, smoking, physical activity, and daily intake of calories, were collected using the standardized questionnaires. Diabetes mellitus was defined as current fasting blood sugar ≥126 mg/dL, or HbA1c ≥ 6.5, or subject’s self-reporting of diabetes mellitus based on a doctor’s diagnosis, or current use of anti-diabetic drugs. Hypertension was defined as current systolic blood pressure ≥ 140 mmHg or diastolic blood pressure ≥ 90 mmHg, or subject’s self-reporting of hypertension based on a doctor’s diagnosis, or current use of anti-hypertension drugs. Smoking refers to current or past use of cigarettes or hookahs or pipes. Hookahs is a single- or multi-stemmed instrument for vaporizing and smoking flavored tobacco, whose vapor or smoke is passed through a water basin-often glass-based-before inhalation. Physical Activity was measured using Aadahl et al. physical activity questionnaire [22]. Daily intake of calories (Kcal) was assessed by a standardized 24-h dietary recall questionnaire.

Anthropometric measurements including height, weight, and waist circumference (WC) as well as laboratory measurements including LDL, HDL, total cholesterol, and triglyceride were performed under standard conditions with calibrated instruments. Anthropometric measurements were taken with shoes removed and the participants wearing light clothing. Height and weight were measured with a fixed stadiometer and a digital scale according to the standard protocol. A flexible, circumference measuring tape is used to measure the waist (WC). WC should be measured at a point midway between the iliac crest and the lowest rib in a standing position [19]. High WC was defined as waist circumference >102 cm in males and >88 cm in females.

An overnight fasting venous blood sample was obtained for every participant for biochemical measurements. A total of 25 ^cc^ of whole blood is collected by a trained nurse. Fasting blood sugar was measured by enzymes (glucose oxidase) colorimetric method using a commercial kit (Pars Azmun, Karaj, Iran). HbA1C was measured by boranate affinity method using a CERA-STAT system (CERAGEM MEDISYS, chungcheongnam-do, Korea). Total cholesterol was measured by enzymatic (cholesterol oxidase phenol aminoantipyrine (CHOD-PAP)) colorimetric method using a commercial kit (Pars Azmun) [19]. High total cholesterol was considered as total cholesterol ≥200 mg/dL. HDL cholesterol was measured by enzymatic (cholesterol esterase and cholesterol oxidase (CHE & CHO)) colorimetric method using a commercial kit (Pars Azmun) [19]. Low HDL cholesterol was defined as high density lipoprotein cholesterol < 40 mg/dL in males, and < 50 mg/dL in females. LDL cholesterol was measured by enzymatic (CHE & CHO) colorimetric method using a commercial kit (Pars Azmun) [19]. High LDL cholesterol was considered as low-density lipoprotein cholesterol ≥110 mg/dL. Triglyceride was measured by enzymatic (glycerol-3- phosphate oxidase phenol aminoantipyrine (GPO-PAP)) colorimetric method using a commercial kit (Pars Azmun) [19]. High serum triglyceride was defined as serum triglyceride ≥150 mg/dL.

### Statistical analysis

In the descriptive analysis, we used the mean (standard deviation), and number (percent) for the continuous and categorical variables respectively. To investigate the association between the risk factors and dependency in IADL and BADL, at first, the directed acyclic graph (DAG) was depicted based on the literature review, considering the activities of daily living as the outcome and the cardio-metabolic and socio-demographic risk factors as the explanatory variables (see Figure S1 in the Additional file 1). We used the DAG to help us choose the proper covariates (confounders, not intermediate or collider variables) to enter into the multiple models. The logistic regression model via Hosmer and Lemeshow suggested strategy [23] was used to investigate the association between BADL and IADL and cardio-metabolic and socio-demographic risk factors based on the causal graph. The risk factors that their effect should be controlled based on the DAG, as well as the *P*-value of their association with the outcome was ≤0.25 in the bivariate analysis, were entered into the multiple logistic model. Then the included risk factors were removed from the model one by one when they lost their significance while checking via a likelihood ratio test. Afterward, the statistical significance of the plausible interaction terms between the remaining risk factors in the model was assessed. We chose the interaction terms based on the literature review and expert opinion. No interaction term was statistically significant. Eventually, the goodness of fit of the final model to the data was checked out using the Hosmer-Lemeshow test. Stata version 15.1 (Stata Corp) was used for statistical analysis.

## Results

Mean (Standard Deviation) of the participants’ age was 69.3 (6.4) years (range: 60 and 96 years). 76.8% of the participants were married. About 67% of the people were literate. The socio-demographic characteristics of the participants were presented in Table 1.

**Table 1:** Participants’ socio-demographic characteristics (*n* = 2426)

Characteristic	No. (%)
Men	1166 (48.1)
*Age (years)*	
60–64	598 (24.7)
65–69	952 (39.2)
70–74	379 (15.6)
75–79	282 (11.6)
80–84	146 (6.0)
≥ 85	69 (2.8)
*Marital status*	
Single	19 (0.8)
Married	1864 (76.8)
Divorced	20 (0.8)
Widow	523 (21.6)
*Education*	
No education	800 (33.0)
Primary School	885 (36.5)
Guidance school	218 (9.0)
High school	332 (3.7)
Academic	189 (7.8)

In all, 1235 out of 2179 (proportion (95% CI); 56.7 (54.6–58.8) %), and 560 out of 2381 of the study participants (proportion (95% CI); 23.5 (21.9–25.3) %) were dependent in the IADL and BADL, respectively.

Mean (Standard Deviation) of the age of dependent and independent people in the IADL were 70.3 (6.9) and 67.7 (5.0) years respectively. Table 2 presents the association between the socio-demographic and cardio-metabolic risk factors with dependency in the IADL using simple and multiple logistic regressions. In Table 2, the distribution of the risk factors according to the IADL status (“dependent” status) has been shown in the column “*Dependent No. (%)*”. Obviously, the “independent” status of the IADL can be reconstructed using the data of the “dependent” status. Column “*Crude OR (95%CI)*” of Table 2 shows the prevalence odds ratio of the association of each risk factor with IADL in the simple logistic regression. Estimation of the prevalence odds ratio of each risk factor was adjusted for other risk factors entered into the multiple logistic regression, and they presented in column “*adjusted OR (95%CI)*” of Table 2. In this column, adjusted ORs have been displayed only for the risk factors that remained in the final multiple logistic model, after fitting the model by the backward method. The *p*-value of Hosmer and Lemeshow goodness of fit (GOF) test for the IADL multiple logistic model (with 8 degrees of freedom) was 0.11.

**Table 2:** Assessment of the association between the cardio-metabolic and socio-demographic risk factors and dependency in the IADL using simple and multiple logistic regressions

Risk Factor		Dependent No. (%)	Crude OR^a^ (95%CI)	*P* value^b^	Adjusted OR^a^ (95%CI)^c^	*P* value^d^
*Age (years)*	60–64	267 (48.5)	1	–	1	–
	65–69	453 (52.3)	1.2 (0.9–1.4)	0.16	1.1 (0.8–1.3)	0.70
	70–74	196 (57.5)	1.4 (1.1–1.9)	0.009	1.3 (1.0–1.7)	0.10
	75–79	172 (70.2)	2.51 (1.8–3.5)	< 0.001	1.9 (1.3–2.7)	< 0.001
	80–84	100 (80.7)	4.43 (2.8–7.1)	< 0.001	3.5 (2.1–5.8)	< 0.001
	≥ 85	47 (90.4)	10.0 (3.9–25.5)	< 0.001	6.6 (2.5–17.3)	< 0.001
*Sex*	Female	817 (69.0)	1	–	1	–
	Male	418 (42.1)	0.3 (0.3–0.4)	< 0.001	0.4 (0.4–0.5)	< 0.001
*Education*	No education	533 (75.1)	1	–	1	–
	Primary School	465 (58. 6)	0.5 (0.4–0.6)	< 0.001	0.6 (0.5–0.8)	< 0.001
	Guidance school	81 (40.9)	0.23 (0.2–0.3)	< 0.001	0.4 (0.3–0.5)	< 0.001
	High school	103 (34.3)	0.17 (0.1–0.2)	< 0.001	0.3 (0.2–0.4)	< 0.001
	Academic	53 (29.9)	0.1 (0.1–0.2)	< 0.001	0.3 (0.2–0. 5)	< 0.001
*Diabetes mellitus*	No	770 (53.4)	1	–	1	–
	Yes	462 (63.0)	1.49 (1.2–1. 8)	< 0.001	1.4 (1.2–1.7)	< 0.001
*Hypertension*	No	294 (50.6)	1	–	–	–
	Yes	941 (58.9)	1.4 (1.2–1.7)	0.001	–	–
*High total cholesterol*	No	825 (56.1)	1	–	–	–
	Yes	409 (57.8)	1.1 (0. 9–1.3)	0.47	–	–
*Low HDL cholesterol*	No	577 (52.2)	1	–	–	–
	Yes	657 (61.2)	1.4 (1.2–1.7)	< 0.001	–	–
*High LDL cholesterol*	No	643 (57.0)	1	–	–	–
	Yes	591 (56. 5)	1.0 (0.8–1.2)	0.83	–	–
*High serum triglyceride*	No	841 (56. 6)	1	–	–	–
	Yes	393 (56.9)	1.0 (0.8–1.2)	0.89	–	–
*Smoking*	Never	525 (53.9)	1	–	1	–
	Past	476 (63.2)	1.5 (1.2–1. 8)	< 0.001	1.3 (1.0–1.6)	0.047
	Current	234 (51.8)	0.9 (0.7–1.2)	0.45	0.8 (0.6–1.1)	0.14
*BMI*	< 18.5	24 (57.1)	1	–	–	–
	18.5–24.9	352 (56.2)	1.0 (0.5–1.8)	0.91	–	–
	25–29.9	496 (53.1)	0.9 (0.5–1.6)	0.61	–	–
	≥30	363 (62.9)	1.3 (0.7–2.4)	0.5	–	–
*High Waist Circumference*	No	417 (56.3)	1	–	–	–
	Yes	818 (56.9)	1.0 (0.9–1.2)	0.8	–	–
*Physical Activity*	No	993 (60.2)	1	–	1	–
	Yes	242 (45.8)	0.6 (0.5–0.7)	< 0.001	0.7 (0.5–0.8)	< 0.001
*Daily intake of calories*		1534.0 (582.4)^e^	.9996 (.9995–.9998)	< 0.001	–	–

a: Prevalence Odds Ratio: They indicate what is the chance of having dependency in the IADL in each group compared to the reference group (first group) of each risk factor?

b: In the bivariate analysis

c: Adjusted OR (95%CI) have been displayed only for the risk factors that remained in the final multiple logistic model, after fitting the model by the backward method. The risk factors “age”, “sex”, “education”, “diabetes mellitus”, “hypertension”, “low HDL cholesterol”, “smoking”, “physical activity” and “daily intake of calories” entered into the first multiple logistic model and after fitting the model by the backward method, the covariates “age”, “sex”, “education”, “diabetes mellitus”, “smoking” and “physical activity” remained in the final model

d: In the multiple logistic regression

e: Mean (Standard Deviation)

Mean (Standard Deviation) of the age of dependent and independent participants in the BADL were 71.3 (7.3) and 68.7 (6.0) years, respectively. The association between the socio-demographic and cardio-metabolic risk factors with dependency in the BADL has been presented in Table 3. In Table 3, the distribution of the risk factors according to the BADL status (“dependent” status) has been shown in the column “*Dependent No. (%)*”. Column “*Crude OR (95%CI)*” of Table 3 shows the prevalence odds ratio of the association of each risk factor with IADL in the simple logistic regression. Estimation of the prevalence odds ratio of each risk factor was adjusted for other risk factors entered into the multiple logistic regression, and they presented in column “*adjusted OR (95%CI)*” of Table 3. In this column, adjusted ORs have been displayed only for the risk factors that remained in the final multiple logistic model, after fitting the model by the backward method. The result of the Hosmer and Lemeshow GOF test for the BADL multiple logistic model (with 8 degrees of freedom) was not statistically significant (*P*-value =0.17).

**Table 3:** Assessment of the association between the cardio-metabolic and socio-demographic risk factors and dependency in the BADL using simple and multiple logistic regressions

Risk Factor		Dependent No. (%)	Crude OR^a^ (95%CI)	*P* value^b^	Adjusted OR^a^ (95%CI)^c^	*P* value^d^
*Age (years)*	60–64	98 (16.6)	1	–	1	–
	65–69	191 (20.5)	1.3 (1.0–1.7)	0.06	1.2 (0.9–1.5)	0.33
	70–74	95 (25.6)	1.7 (1.3–2.4)	0.001	1.6 (1.1–2.2)	0.007
	75–79	101 (36.3)	2.9 (2.1–4.0)	< 0.001	2.4 (1.7–3.4)	< 0.001
	80–84	39 (27.9)	1.9 (1.3–3.0)	0.003	1.5 (1.0–2.4)	0.078
	≥ 85	36 (52.2)	5.5 (3.3–9.2)	< 0.001	4.0 (2.3–7.0)	< 0.001
*Sex*	Female	405 (32.9)	1	–	1	–
	Male	155 (13.5)	0.3 (0.3–0.4)	< 0.001	0.4 (0.3–0.5)	< 0.001
*Education*	No education	268 (34.4)	1	–	1	–
	Primary School	204 (23.3)	0.6 (0.5–0.7)	< 0.001	0.8 (0. 7–1.1)	0.154
	Guidance school	37 (17.2)	0.4 (0.3–0.6)	< 0.001	0.8 (0.5–1.2)	0.20
	High school	35 (10.7)	0.2 (0.2–0.3)	< 0.001	0.5 (0.3–0.7)	< 0.001
	Academic	16 (8.6)	0.2 (0.1–0.3)	< 0.001	0.5 (0.3–0.8)	0.008
*Diabetes mellitus*	No	355 (22.4)	1	–	–	–
	Yes	204 (25.8)	1.2 (1.0–1.5)	0.07	–	–
*Hypertension*	No	125 (19.5)	1	–	–	–
	Yes	435 (25.0)	1.4 (1.1–1.7)	0.005	–	–
*High total cholesterol*	No	367 (23.0)	1	–	–	–
	Yes	192 (24.5)	1.1 (0.9–1.3)	0.43	–	–
*Low HDL cholesterol*	No	253 (20.8)	1	–	1	–
	Yes	306 (26.3)	1.4 (1.1–1.7)	0.001	1.2 (1.0–1.5)	0.08
*High LDL cholesterol*	No	289 (23.6)	1	–	–	–
	Yes	269 (23.3)	1.0 (0.8–1.2)	0.89	–	–
*High serum triglyceride*	No	370 (22.8)	1	–	–	–
	Yes	189 (25.0)	1.1 (0.9–1.4)	0.22	–	–
*Smoking*	Never	242 (22.9)	1	–	–	–
	Past	205 (24.7)	1.1 (0.9–1.4)	0.36	–	–
	Current	113 (22. 8)	1.0 (0.8–1.3)	0.95	–	–
*BMI*	< 18.5	12 (25.5)	1	–	–	–
	18.5–24.9	137 (19.7)	0.7 (0.4–1.4)	0.33	–	–
	25–29.9	214 (21.3)	0.8 (0.4–1.6)	0.49	–	–
	≥30	197 (31.1)	1.3 (0.7–2.69)	0.42	–	–
*High Waist Circumference*	No	174 (21.2)	1	–	–	–
	Yes	386 (24.8)	1.2 (1.0–1.5)	0.05	–	–
*Physical Activity*	No	490 (26.6)	1	–	1	–
	Yes	70 (12.9)	0.4 (0.3–0.5)	< 0.001	0.5 (0.4–0.6)	< 0.001
*Daily intake of calories*		1414.5 (533.5) ^e^	0.99 (0.99–0.99)	< 0.001	0.99 (0.99–0.99)	0.001

a: Prevalence Odds Ratio: They indicate what is the chance of having dependency in the BADL in each group compared to the reference group (first group) of each risk factor?

b: In the bivariate analysis

c: Adjusted OR (95%CI) have been displayed only for the risk factors that remained in the final multiple logistic model, after fitting the model by the backward method. The risk factors “age”, “sex”, “education”, “diabetes mellitus”, “hypertension”, “low HDL cholesterol”, “high serum triglyceride”, “high waist circumference” “physical activity” and “daily intake of calories” entered into the first multiple logistic model and after fitting the model by the backward method, the covariates “age”, “sex”, “education”, “low HDL cholesterol”, “physical activity” and “daily intake of calories” remained in the final model

d: In the multiple logistic regression

e: Mean (Standard Deviation)

## Discussion

The goal of our study was to assess the association between cardio-metabolic and socio-demographic risk factors with BADL and IADL in older people. The study results showed that 56.7 and 23.5% of the participants were dependent on the IADL and BADL respectively. After adjusting for potential confounders, being older and female, having a lower education level, a history of diabetes mellitus and past smoking, and no physical activity were significantly associated with dependency in the IADL. Also, being older and female, having a lower education level, no physical activity and daily intake of calories were associated with dependency in the BADL.

In a study in Poland, Ćrwirlej-Sozańska et al. reported the lower dependency in the IADL and BADL (35.8 and 17.1%, respectively) than our study [24]. Also, the percentages of the dependency in our study were much higher than those observed in the studies in Ireland [25] and Nepal [26], but lower than in Panama [27]. Diversities in the prevalence of dependency among different countries can be due to differences in the frequency of its various risk factors in those communities.

In a study in the Netherlands, age was reported as one of the most important predictors of disability [9]. In another study conducted in 6 middle and low-income countries, older age, were also identified as the important factor affecting disability [10]. Our study results were in agreement with these results.

In most studies, being a woman has been mentioned as one of the predictors of disability due to several reasons including higher life expectancy, lower-income, and physical weakness [8, 24]. However, in a study in Panama, being male increased the odds of disability in IADL and BADL, with the possible causes being lower smoking for women and equal monthly income in both sexes [27]. In our study, being female was a risk factor for dependency in both IADL and BADL.

Chronic illness increases the chance of developing disability [24]. According to the study by Marengoni et al. in Sweden, almost none of those without chronic disease were dependent on BADL. The incidence of disability was lowest in people with CVD and highest in those with mental and cerebrovascular diseases [28]. In a study, in diabetic patients, BMI along with cardio-metabolic risk factors (hypertension, history of CVD, impaired eGFR, TG, and HDL cholesterol) explained 25–35% excess odds of disability. In that study, diabetes mellitus increased 2-fold the risk of disability [29]. Given that diabetes is associated with neuropathy, retinopathy, and PAD, such a relationship is not unexpected. In another study in Australia, disability was associated with smoking (OR: 1.8 (1.2–2.78)), obesity (OR: 3.0 (1.8–4.8)), and diabetes (OR: 2.0 (1.1–3.5)) [8]. Our study results showed that diabetes mellitus and past smoking were associated with dependency in the IADL. A possible reason for the not seeing of association between other chronic cardiovascular diseases and older adults’ dependency in our study was the simultaneous control of their confounding effects in the models.

In a review study [30], a disagreement between prospective and experimental studies was shown in the effect of late-life physical activity on minimizing functional disability. Several well-conducted prospective studies show a beneficial effect of physical activity on minimizing disability, whereas the majority of experimental studies that have examined disability as an outcome do not show improvements in disability. Our study showed that no physical activity was a risk factor for dependency in both IADL and BADL, which can be resulting from inverse causation bias due to cross-sectional data.

It is worth mentioning that disability and care dependency are not completely interchangeable terms. Dependency in BADL can provide a proxy of disability, but this is not completely the case for IADL. One of the limitations of our study is that the analysis is based only on data from cardio-metabolic and socio-demographic risk factors, while much of the association between illness and dependency is due to synergistic effects [31]. The second limitation of the present study is due to the design of our study, which is cross-sectional and cannot confirm the temporal priority of the risk factors and dependency. For example, the association of the dependency in IADL and BADL with decreased physical activity may be justified by the fact that people with disabilities in one domain are more likely to have limitations for having physical activity. This phenomenon is called inverse causation. Another limitation of the study is that although the BEH study is a population-based cohort, it does not include people residing in care facilities. So, the percentage of people with IADL and BADL disability may be higher than it was observed in this study. Therefore, when interpreting and using the results of this study, the above points should be considered.

## Conclusion

After adjusting for other potential cardio-metabolic and socio-demographic confounders, being older and female, having a lower education level and no physical activity are the cardio-metabolic and socio-demographic risk factors that increase the odds of being dependent in both of the BADL and IADL in the older adults. A history of diabetes mellitus and past smoking increases only the odds of being dependent on the IADL, and a high daily intake of calorie is the risk factor that increases just the odds of being dependent on the BADL. Dependency in older adults can be prevented by increasing community literacy, improving physical activity, preventing and controlling diabetes mellitus, avoiding smoking, and reducing daily calorie intake. In order to confirm the above results, other studies are proposed in which other confounding factors are also controlled, and the temporal priority of risk factors on dependency is clear. The results of this study can be used for the practice of geriatrics and health care of older adults.

## Supplementary Information

**Additional file 1: Figure S1.** DAG of cardio-metabolic/socio-demographic risk factors and BADL/IADL.

## Data Availability

The data that support the findings of this study are available from the principal investigator of Bushehr Elderly Health (BEH) program but restrictions apply to the availability of these data, which were used under license for the current study, and so are not publicly available.

## Abbreviations

BEH: Bushehr Elderly Health
BADL: Basic Activities of Daily Living
IADL: Instrumental Activities of Daily Living
OR (95%CI): Odds Ratio (95% Confidence Interval)
ICF: International Classification of Functioning, Disability, and Health
ADL: Activities of Daily Living
GBD: Global Burden of Disease
BMI: Body Mass Index
WC: Waist Circumference
CHOD-PAP: Cholesterol Oxidase Phenol Aminoantipyrine
CHE & CHO: Cholesterol Esterase and Cholesterol Oxidase
GPO-PAP: Glycerol-3- Phosphate Oxidase Phenol Aminoantipyrine
HRs: Hazard Ratios
CVD: Cardiovascular Diseases
MI: Myocardial Infarction
DALY: Disability Adjusted Life Years
YLL: Years of Life Lost
YLD: Years lived with disability
LDL: Low Density Lipoprotein
HDL: High Density Lipoprotein
DAG: Directed Acyclic Graph
GOF: Goodness of Fit
eGFR: Estimated Glomerular Filtration Rate
TG: Triglyceride
PAD: Peripheral Artery Disease
