# Supplementary figures and images for: Cardio-metabolic and socio-demographic risk factors associated with dependency in basic and instrumental activities of daily living among older Iranian adults: Bushehr elderly health program
Source: BMC Geriatr. 2021 Mar 9;21:172. doi: 10.1186/s12877-021-02124-x (PMC7941716; doi:10.1186/s12877-021-02124-x)

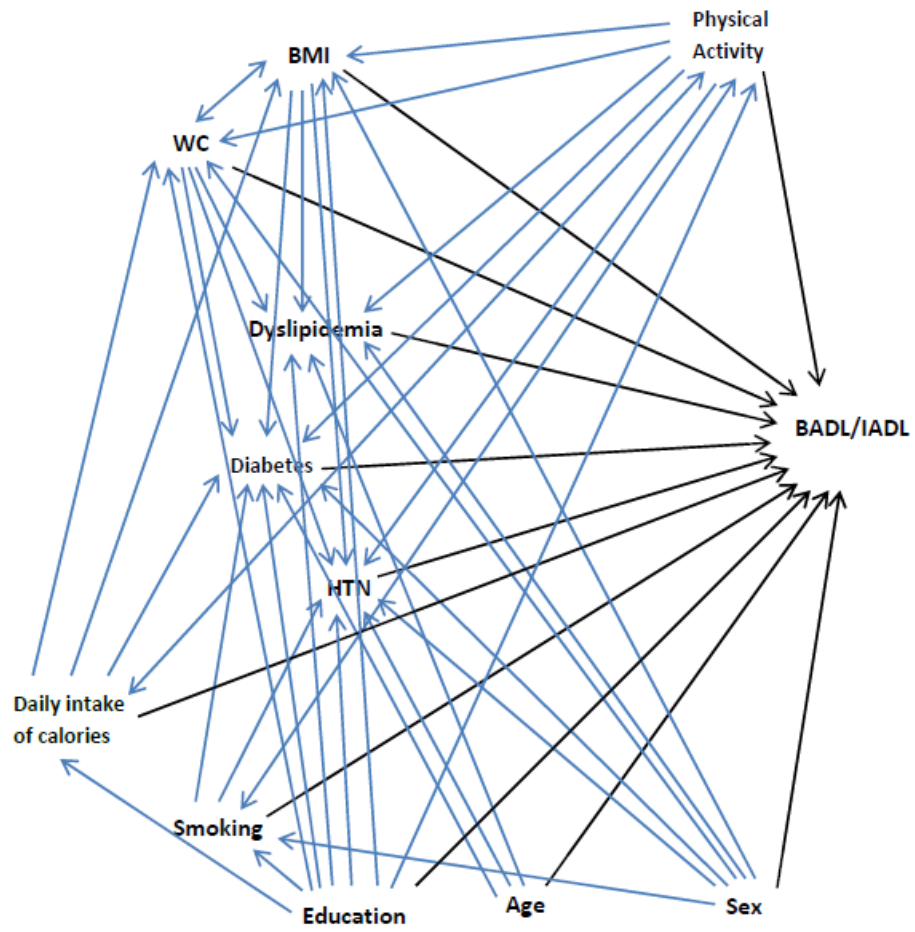

**Figure 1:** DAG of cardio-metabolic/socio-demographic risk factors and BADL/IADL

Supplement: Supplementary file 1 — Additional file 1: Figure S1. DAG of cardio-metabolic/socio-demographic risk factors and BADL/IADL. [file 12877_2021_2124_MOESM1_ESM.pdf]
